# A Photothermal-Responsive Soft Actuator Based on Biomass Carbon Nanosheets of Synergistic Bilateral Polymers

**DOI:** 10.3390/polym16243476

**Published:** 2024-12-13

**Authors:** Jianze Chen, Quanzhong Wei, Honglin Wang, Wenjia Cui, Xuewei Zhang, Yuanyuan Wang

**Affiliations:** 1 Key Laboratory of Advanced Materials of Tropical Island Resources of Ministry of Education, School of Materials Science and Engineering, Hainan University, Haikou 570228, China; chjzemail@163.com (J.C.); 23220856010011@hainanu.edu.cn (Q.W.); 19935342032@163.com (W.C.); 2 School of Tropical Agriculture and Forestry, Hainan University, Haikou 570228, China; wanghonglin0708@163.com; 3 NHC Key Laboratory of Tropical Disease Control, School of Tropical Medicine, Hainan Medical University, Haikou 571199, China

**Keywords:** photothermal conversion, hydrogel, polymer, actuator

## Abstract

Currently, polymer actuators capable of photothermal response are being developed to be more sensitive and repeatable. In this work, a three-layered structured soft film actuator (NA/PET/NI-3) was designed by combining poly(N-isopropylacrylamide) (PNIPAM), poly(N-(2-aminoethyl)-acrylamide) (PANGA) and poly(ethylene glycol-co-terephthalate) (PET) film. Coconut water and PEI were used to synthesize a new kind of carbon nanosheet (PEI-CCS), which, when triggered by near-infrared light, will enable photothermal bending behavior in the micrometer-scale NA/PET/NI-n film, while PET served as the supporting and heat conducting layer. This three-layered actuator utilized the synergistic effects of two kind of polymers, PNIPAM and PNAGA, on either side of PET, with the upper critical solution temperatures and lower critical solution temperatures when subjected to temperature changes. This bilateral polymer design exhibited a rapid response under near-infrared light stimulation, bending to 180° within 4 s and recovering to its original shape within 30 s. When the bending process was set to 90° as in the standard experiment, NA/PET/NI-3 responded within 2 s and recovered within 8 s. NA/PET/NI-3 also demonstrated good reversibility and repeatability, capable of undergoing reversible driving over 120 times. The design and preparation of this actuator provided new ideas for the development of polymer soft actuators.

## 1. Introduction

Smart hydrogels regulated by polymer composition make highly attractive components for the development of soft actuators, wearable technology, and healthcare devices. In particular, stimulus-responsive hydrogels can perceive and activate their intelligent functions in the presence of various stimuli. In recent decades, hydrogels have evolved to respond to a wide range of stimuli, leading to macroscopic deformation and actuation [1,2,3]. Light is a practical external stimulus source that can remotely induce volume changes in photoresponsive polymers under illumination. Using localized photothermal heating, the reversible dehydration/hydration process of photoresponsive hydrogels can be induced [4,5]. Incorporating photothermal conversion, nanomaterials can accelerate and enhance the dehydration/hydration process. These added photothermal conversion nanomaterials can quickly convert light irradiation into thermal energy, causing localized dehydration and consequently the deformation of the photothermal-responsive hydrogels [6]. 

Over the past twenty years, various nanomaterials with efficient photothermal conversion capabilities, including inorganic nanomaterials (such as gold, neodymium oxide and carbon-based materials) and organic compounds (such as black phosphorus, quinoline blue and porphyrin), have been incorporated into hydrogels to improve the light response rate of the prepared soft actuators [7,8]. Biomass material is energy-efficient raw material for preparing carbon nanomaterials without environment pollution, and the photothermal conversion performance can be controllably improved by introducing nitrogen heteroatom [9,10]. Coconut water is a source of sugars, minerals, and small amounts of vitamins and amino acids [11,12]. A wealth of functional groups regulated by crosslinking can be used to synthesize carbon nanomaterials through bottom-up synthesis methods. PEI is commonly used in polymer design and regulation to introduce nitrogen-containing groups, which can enhance photothermal conversion during the synthesis of carbon nanomaterials [13]. The development of new biomass-based photothermal materials accelerates the photothermal conversion behavior of photothermal response polymer networks, which is crucial for the design of polymer actuators. Moreover, by designing the overall structure of photothermal-responsive hydrogels, not only simple bending and twisting behaviors can be achieved, but also more complex shape transformations can be achieved at a macroscopic scale [14]. For the design of three-dimensional structures of hydrogel actuators, the synthesis of anisotropic structures is fundamental, which is mainly focused on layered structures nowadays. To achieve fast, sensitive, and adjustable actuation, a typical layered hydrogel actuator consists of a two-layered structure that responds asymmetrically under external stimulus conditions [15]. The polymer networks with the upper critical solution temperature (UCST) or the lower critical solution temperature (LCST) are used to fabrication for the anisotropy of the polymer driver [16,17]. However, it is very difficult to synergistically utilize the advantages of the bilateral polymers due to the influence of the interface, and the size in the millimeter range at a macro scale may limit the practical application of the actuator.

Poly(N-isopropylacrylamide) and poly(N-(2-aminoethyl)-acrylamide are typical temperature-responsive polymers [18,19]. The two polymers have opposite thermal response swelling and shrinking properties at low and high temperatures [20,21]. For the design of polymer actuators, the addition of photothermal nanoparticles has the advantages of remote control, low invasiveness and high spatiotemporal selectivity [22]. In this contribution, the water of Wenchang coconuts and polyethyleneimine were used as carbon sources to prepare CS by bottom-up synthesis using a simple microwave method. Based on the photothermal conversion function of CS, we combined PNIPAM and PANGA, two different polymers, using polyethylene terephthalate (PET) film as a barrier which could conduct heat between two layers of polymer. Our strategy may provide new insights into the design of layered-structure actuators. 

## 2. Materials and Methods

### 2.1. Materials

Fresh Wenchang coconuts were supplied by the school of Tropical Agriculture and Forestry, Hainan University. Polyethyleneimine (Mw = 1800) and N,N′-methylenebisacrylamide (MBAA) were purchased from Shanghai Aladdin Bio-chem Technology Co., Ltd. (Shanghai, China) PET film (thickness: 12.5 µm) was purchased from Suzhou Dongxuan Plastic products Co., Ltd. (Suzhou, China), N-isopropylacrylamide (NAGA) was purchased from Tokyo Chemical Industry Co. (TCL, Shanghai, China), N-(2-aminoethyl)-acrylamide (NIPAM) was purchased from Shanghai Adamasi Reagents Co., Ltd. (Shanghai, China), and 2-hydroxy-4′-(2-hydroxyethoxy)-2-methylpropiophenone (Irg. 2959) was purchased from Bide Pharmatech Ltd. (Shanghai, China).

### 2.2. Preparation of PEI-CCS

The polyethyleneimine–coconut water carbon nanosheets (PEI-CCS) were synthesized via a simple microwave method. In detail, coconut water was filtered through a membrane with a pore size of 20 μm. An amount of 1 g PEI was dissolved in 200 mL purified coconut water, followed by microwave treatment in a microwave oven at 700 watts. The microwave carbonization time was adjusted to 3 min, 3.5 min, and 4 min, respectively, to prepare three different PEI-CCS. Then, the products were collected after microwaving with deionized water to obtain a uniform PEI-CCS solution.

### 2.3. Preparation of NA/PET/NI-n Soft Actuator

To prepare 1 M NIPAM solution, 339.4 mg NIPAM monomer was dissolved in 3 mL PEI-CCS solution. Then, 0.1 M of chemical crosslinker MBAA and 1.0 M initiator Irg. 2959 were added in the mixture solution for PNIPAM layer spin coating. To prepare 4.0 M NAGA monomer solution, 512.5 mg of NAGA was dissolved in 1 mL of deionized water. After that, 0.4 M of chemical crosslinker MBAA and 4.0 M initiator Irg. 2959 were added in the solution for PNAGA layer spin coating. The PET film was cut into square pieces of 40 × 40 mm^2^, then placed in a plasma cleaner for surface treatment for 60 min. The PET film edges were fixed onto a 60 × 60 mm^2^ glass slide using waterproof tape. Two hundred microliters NAGA prepolymer solution was placed drop-wise onto the surface of the PET film on a spin coater with a rotation speed of 1000 rpm, an acceleration of 500 rpm·s^−1^ and a spin time of 60 s. The spin-coated sample was then placed under a xenon lamp light source, equipped with a 365 nm wavelength filter to initiate polymerization. The photoinitiation time was set to 1 minute to make one layer. To increase the thickness of the layer, the steps of solution application, spin coating, and photoinitiation were repeated. The repeated times of PNAGA polymerization were defined as *n* times. The prepared PET-PNAGA bilayer film was peeled off from the glass slide and flipped over. Similarly, the edges were again fixed onto the glass slide using waterproof tape, then 200 µL of NIPAM prepolymer solution was placed drop-wise onto the surface with a rotation speed of 1000 rpm, an acceleration of 500 rpm·s^−1^, and a spin time of 60 s. The spin-coated sample was polymerized under the xenon lamp light source with the photoinitiation time set to 2 min. The repeated times of PNIPAM polymerization were defined as m times, m plus n equals 10 layers. By regulating the number of spin-coated layers of the PNAGA hydrogel and the PNIPAM hydrogel, five different hydrogel thickness ratios of soft actuators (NA/PET/NI-n) were obtained, which were denoted as NA/PET/NI-1, NA/PET/NI-2, NA/PET/NI-3, NA/PET/NI-4 and NA/PET/NI-5, respectively. The ambient temperature was maintained at 25 °C, with a relative humidity of 73 ± 3% RH.

### 2.4. Characterization

To prepare the sample for transmission electron microscopy analysis, 50 µL of PEI-CCS solution of PEI-CCS solution was placed drop-wise onto an ultrathin carbon film and left to dry at room temperature in a ventilated environment for 24 h until completely dry. After drying, the TEM images of the PEI-CCS were observed using a field emission transmission electron microscope (Talos F200X G2, Thermo Fisher Scientific, Shanghai, China).

To observe atomic force microscopy, a drop of PEI-CCS solution was placed on a flat silicon wafer. After drying, the sample was placed into the AFM sample chamber, and the morphological structure of the PEI-CCS nanomaterials was observed by atomic force microscopy (BRUKER, Berlin, Germany). 

Fourier transform infrared spectrometry was performed on a FTIR spectrometer (T27, BRUKER, Germany) with the KBr pellet method. The wavelength range for testing was set from 4000 to 500 cm^−1^.

To investigate the ultraviolet spectroscopy (UV) of PEI-CCS, the solution was poured into a clean cuvette, and the absorption of the PEI-CCS solution at 808 nm was measured by an ultraviolet spectrophotometer (UV-2600i, Shimadzu Instrument, Suzhou, China).

To investigate photothermal conversion efficiency, a simple method was used. In detail, 5 mL of PEI-CCS solution was placed in a transparent sample vial, and the top of the solution was irradiated with a near-infrared laser (DG73139, CNI, Changchun, China). The laser power was set to 5000 mW, with a wavelength of 808 nm, and the distance between the light source and the liquid surface was maintained at 3 cm. A thermal imaging camera (TiS20+ MAX, FLUKER, Shanghai, China) was used to observe and record the temperature changes in the solution.

To perform scanning electron microscopy (SEM), the prepared actuator sample was immersed in liquid nitrogen for 10 min. The frozen samples were fractured to observe the cross-sectional morphology using a field emission scanning electron microscope (Verios G4 UC, Thermo Fisher Scientific).

The XPS of PEI-CCS and CCD was also investigated using a Axis Supra X-ray photoelectron spectrometer (Shimadzu Kratos, Kyoto, Japan), where the samples of CCD and PEI-CCS were dropped onto a sheet of glass and dried before obtaining the XPS spectra.

Mechanical property tests were conducted using a universal testing machine (Instron 5944 Micro Tester, Norwood, MA, USA). PET films and NA/PET/NI-3 samples were cut into rectangular shapes (5 × 30 mm^2^). Under the tensile mode, the test speed was set to 10 mm min^−1^. For the PET samples, after clamping, the dimensions were 20.0 mm × 5.0 mm × 12.5 μm (length × width × thickness), while for the NA/PET/NI-3 samples, the dimensions were 20.0 mm × 5.0 mm × 38.5 μm (length × width × thickness), with a thickness of 38.5 μm calculated based on SEM data. Tensile strength tests were performed on all samples using a 2 kN sensor, and each experiment was repeated three times.

To evaluate the actuation performance of the prepared hydrogel actuator, the prepared samples was cut into a rectangular shape with dimensions of 5 × 20 mm^2^, and irradiated using a near-infrared (NIR) laser (DG73139, CNI, Changchun, China). The laser power was set to 5000 mW, and the wavelength of the NIR light was 808 nm. The distance between the light source and the actuator was maintained at 3 cm. During NIR laser irradiation, an optical camera was used to record the actuation response of the soft actuator. The ambient temperature was maintained at 25 °C, with a relative humidity of 73 ± 3% RH.

## 3. Results and Discussion

### 3.1. The Synthesis of PEI-CCS

**Figure 1:**
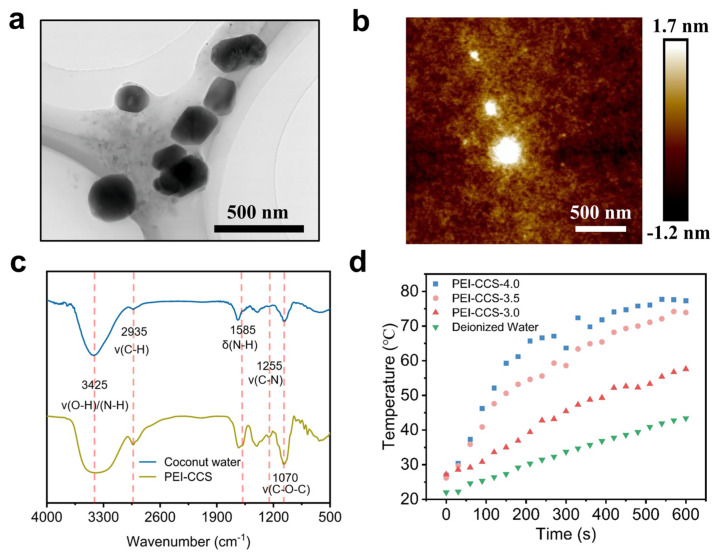
The synthesis of PEI-CCS carbon nanosheets. (**a**) TEM image of PEI-CCS after a microwave carbonization time of 3.5 min. (**b**) AFM image of the PEI-CCS solution after a microwave carbonization time of 3.5 min. (**c**) Fourier transform infrared (FTIR) spectra of coconut water and PEI-CCS. (**d**) The temperature–time variation curves of deionized water and PEI-CCS solution with a carbonization time of 3.0, 3.5 and 4.0 min under near-infrared light irradiation.

To observe the morphology of the prepared PEI-CCS, the particle diameter of PEI-CCS was tested using transmission electron microscopy. The microwave carbonization time was adjusted to 3.0, 3.5, and 4.0 min, respectively, to prepare three different PEI-CCS. As shown in Figure 1a, when the microwave carbonization time was set as 3.5 min, the diameter of the PEI-CCS ranged between 200 and 300 nm. To further determine the three-dimensional structure of PEI-CCS, an atomic force microscope was employed to characterize the morphology of the PEI-CCS. As illustrated in Figure 1b, the thickness of the PEI-CCS carbon nanomaterials was observed to range from 1 to 3 nm. Combining the results of TEM images, it could be inferred that polyethyleneimine–coconut water carbon material displays a disc-shaped morphology, with a central elevation and lower periphery. After inducing PEI, nanosheets were formed instead of carbon dot materials which were synthesized from coconut water under the same conditions, as shown in Figure S1. In addition, after the introduction of PEI in the nanomaterial, there was no significant change in the infrared spectrum of coconut water. The results of Figure 1c revealed that the peaks for PEI-CCS were largely consistent with those of coconut water because of similar chemical composition. Notably, the peaks at 1585 and 1255 cm^−1^ in the PEI-CCS sample might result from the introduction of nitrogen-containing groups of PEI. 

The absorbance of PEI-CCS solutions at a wavelength of 808 nm was also measured to compare the absorption capacity of near-infrared light (Figure S2). With the increase in the microwave carbonization time, the absorption capacity at 808 nm increased significantly. To further investigate the photothermal conversion efficiency, PEI-CCS solutions with different carbonization times were placed under near-infrared laser. The temperature changes in the solution were observed and recorded using a thermal imaging camera every 30 s for a total of 10 min (Figure 1d). Over 10 min, the temperature of pure water increased by 21 °C, while the solution carbonized all exhibited improved photothermal conversion efficiency, especially in the first 200 s. After near-infrared light irradiation of 10 min, the PEI-CCS carbonized for 3.0 min increased by 30.4 °C, the PEI-CCS carbonized for 3.5 min increased by 47.7 °C and the PEI-CCS carbonized for 4.0 min increased by 50.3 °C. It was obvious that the superior photothermal conversion ability was conducive to the preparation of polymer drivers, while the uniformity of particles decreased with longer carbonization times. Therefore, a microwave carbonization time of 3.5 min was selected for preparing the following hydrogel actuators.

**Figure 2:**
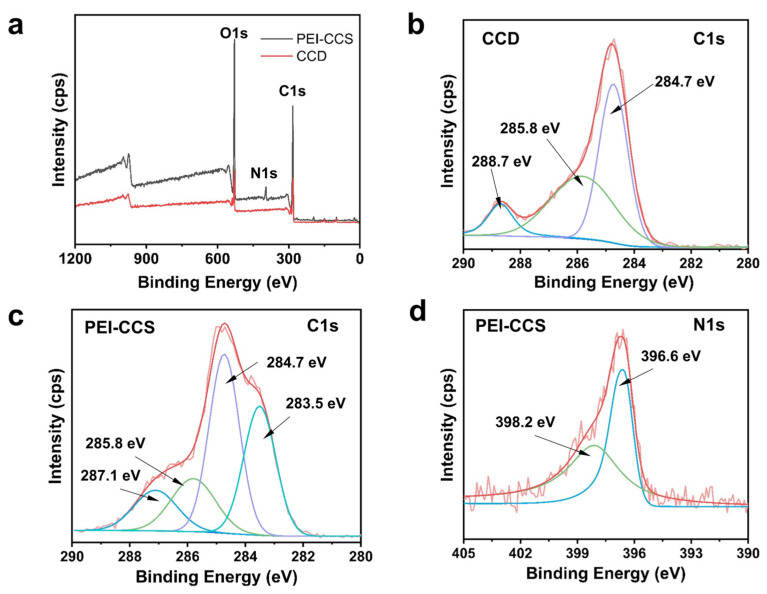
The XPS of PEI-CCS and CCD. (**a**) The wide scan XPS spectra of PEI-CCS and CCD. (**b**) C1s spectra of CCD. (**c**) C1s spectra of PEI-CCS. (**d**) N1s spectra of PEI-CCS.

The amino groups of PEI can chemically react with carboxyl, hydroxyl and other groups in biomass materials to form a crosslinked structure. To explore the nitrogen incorporation function of PEI-CCS, the XPS of PEI-CCS and CCD were also investigated using a Axis Supra X-ray photoelectron spectrometer. As shown in Figure 2a, the results showed that N1s signals are hardly found in the spectra of CCD, whereas the spectra of PEI-CCS indicated an intensified N1s peak. The occurrence of N1s signals of PEI-CCS indicated the introduction of PEI. As shown in Figure 2b, the C1s spectrum of CCD was fitted with the peaks at 284.7, 285.8 and 288.7, and corresponded to C-C, C-O, O=C-O, respectively. For PEI-CCS samples, the C1s band was deconvoluted into four peaks, which correspond to sp2 carbons (C=C, 283.5 eV), sp3 carbons (C−C, 284.7 eV), sp3 carbons (C−O, 285.8 eV) [23,24,25]. The C-N binding peak at 287.1 eV was also observed, suggesting the successful grafting of PEI (Figure 2c). The N1s spectrum was fitted with two peaks at 398.2 and 396.6 eV (Figure 2d). The peak at 396.6 eV corresponded to the nitrogen atoms in PEI, and the peak at 398.2 eV corresponded to the amide CONH [26,27]. These results confirmed that the organic acid groups in CCD turned into amide CONH after introducing PEI and C=C formed after being carbonized and crosslinked with PEI. Microwave carbonization is a process of crosslinking and reconfiguration. On the one hand, the amide bond of PEI is crosslinked with organic acids in coconut water. On the other hand, the C=C sp2 structure generated by the carbonization process is reconstructed to form the carbon nanosheet structure. The nitrogen incorporation aids in forming nanosheets by forming amide bonds, ranging from carbon dots to disc-shaped morphology.

### 3.2. The Design of NA/PET/NI Actuators

**Scheme 1:**
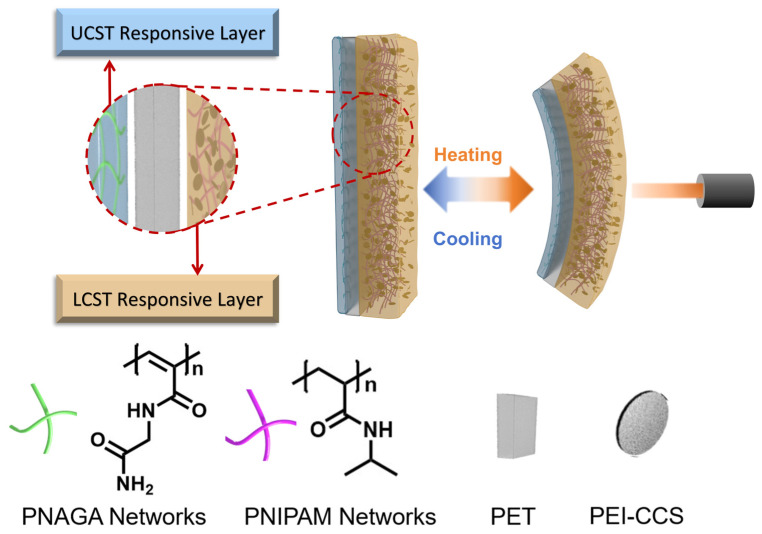
The photothermal response mechanism of the NA/PET/NI-3 soft actuator.

The poly(N-isopropylacrylamide) (PNIPAM) polymer is a typical LCST (lower critical solution temperature) network, which is commonly used in the design of polymer actuators [28,29,30]. However, the driving effect of a single LCST layer is limited. The introduction of UCST (upper critical solution temperature) polymers such as poly(N-(2-aminoethyl)-acrylamide) (PANGA) [31,32] is also required. If a supporting material is selected as a barrier, which can also conduct heat, the introduction of two different polymers on both sides of the supporting layer has the potential to significantly improve the driving effect. The design of bilateral polymer provides a new idea for development of actuator materials. When temperature stimulation occurs, the shrinking of the LCST network and swelling of the UCST network occur simultaneously, and the direction of the two opposite polymers is the same. In this contribution, we developed a new tri-layer soft actuator film NA/PET/NI-3 (Scheme 1) based on PEI-CCS, using PNIPAM as the LCST gel layer, N-(2-aminoethyl)-acrylamide (NAGA) monomer to fabricate the UCST gel layer, and poly(ethylene glycol-co-terephthalate) (PET) as the supporting layer with a thermal conductive function. When the temperature rose above the response temperature, the PET film could conduct heat between the polymers on both sides. When irradiated by near-infrared light, PNIPAM was first heated due to the mixture of PEI-CCS. However, relative to the air layer, the PET film and the PNAGA layer had an insulation effect for the PNIPAM hydrogel, and the bending behavior occurred away from the light source. Similarly, the PNAGA layer also bent away from the light source due to swelling of the segment. This synergistic design of bilateral polymer could lead to more efficient and faster driving behavior. 

To maximize the synergistic driving effect of PNAGA and PNIPAM, it was necessary to optimize the fabrication process of the NA/PET/NI-n composite film. Above all, the overall thickness of actuator significantly affected the driving performance. When the thickness of the PET layer was fixed at 12.5 μm, the total thickness of the polymer film on both sides had an optimal number of layers which could be adjusted by the spin-coating method. By controlling the number of spin-coated layers of the NAGA hydrogel, five different thickness ratios of NA/PET/NI-n actuators were prepared, which were marked as NA/PET/NI-1, NA/PET/NI-2, NA/PET/NI-3, NA/PET/NI-4, and NA/PET/NI-5, respectively.

### 3.3. The Photothermal Response Properties of NA/PET/NI Actuators

**Figure 3:**
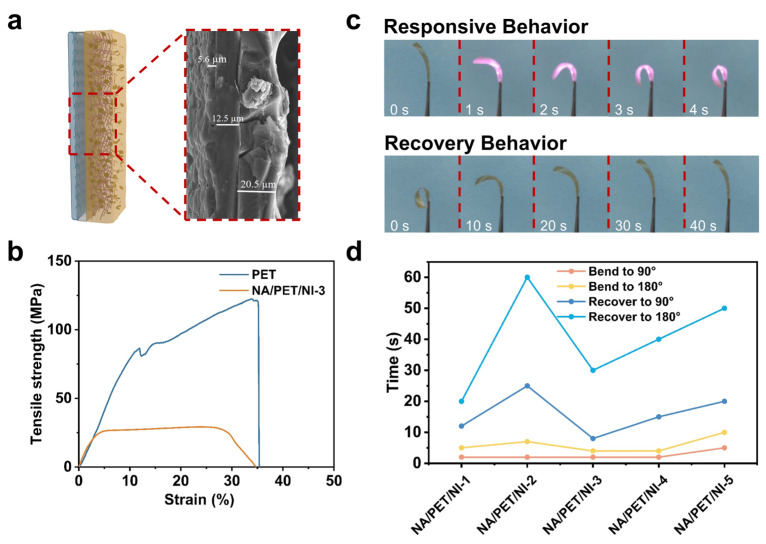
The synthesis and photothermal response properties of the NA/PET/NI-3 soft actuator. (**a**) An illustration and SEM image of the section of the NA/PET/NI-3 soft actuator. (**b**) The stress–strain curves of the PET film and the NA/PET/NI-3 soft actuator. (**c**) The responsive and recovery behavior of NA/PET/NI-3 actuator. (**d**) The actuation performance of soft actuators with different thicknesses.

To investigate the microstructure of the fabricated NA/PET/NI-3 soft actuator, the cross-section of NA/PET/NI-3 was investigated using a scanning electron microscope as shown in Figure 3a. The thickness of the supporting and thermal conductive PET in the middle of actuator was 12.5 μm. The thinner layer, with a thickness of approximately 5.6 µm, was composed of three spin-coated layers of PNAGA hydrogel, while the thicker layer, consisting of seven spin-coated PNIPAM layers, measured a thickness of approximately 20.5 µm. Overall, the actuator had a total thickness of approximately 38.5 µm. Toughness as a mechanical property is the prerequisite for constructing practical actuators. To investigate the mechanical properties of the fabricated NA/PET/NI-3, tensile tests were conducted on both the PET films and NA/PET/NI-3 films. As shown in Figure 3b, the tensile fracture strength of the PET film was 105.06 ± 12.33 MPa, whereas the NA/PET/NI-3 film exhibited a tensile fracture strength of 23.60 ± 4.2 MPa. By calculating the area under the stress–strain curve from the tensile tests, the corresponding fracture energy was also determined. As illustrated in Figure S3, the fracture energy of the PET film was 2331.37 ± 1183.40 J m^−3^, while the fracture energy of the NA/PET/NI-3 film was 400.50 ± 200.07 J m^−3^. These results were consistent with previous results of fracture strength. Although strength and toughness were reduced, the tensile fracture strength was still higher than 23.0 MPa. The strong mechanics of NA/PET/NI-3 would ensure the reciprocating driving behavior in practical applications. In addition, the photothermal response capacity of the prepared actuators under NIR light was recorded using an optical camera. Under near-infrared (NIR) laser irradiation, an optical camera was used to record the photothermal response behavior, and then the NIR light source was removed and the optical camera continued recording the recovery behavior of the soft actuator. As shown in Figure 3c, the NA/PET/NI-3 soft actuator exhibited a rapid response to NIR light, bending to 180° within 4 s and recovering to its original shape within 30 s. The NA/PET/NI-n soft actuators with different thickness ratios were cut into 5 × 20 mm^2^ rectangles and the distance between the light source and the soft actuator was controlled at 3 cm. The response and recovery time of the actuator were observed. As shown in Figure 3d, the NA/PET/NI-3 samples, in which the actuator consisted of three layers of PNAGA and seven layers of PNIPAM, showed the best response sensitivity under conditions of bending and recovering at 90° or 180°. When the bending process was set to 90° as in the standard experiment, NA/PET/NI-3 responded within 2 s and recovered within 8 s. When the bending process was set to 180°, NA/PET/NI-3 responded within 4 s and recovered within 30 s. Therefore, the NA/PET/NI-3 actuator was selected as a proof of concept for demonstrating the following driving properties. The response sensitivity of all bilateral polymers was significantly better than that of a single polymer layer, bending to 90° within 60 s (Figure S4), and this might be attributed to the synergy of the LCST and UCST networks.

### 3.4. The Application of the NA/PET/NI-3 Actuator

**Figure 4:**
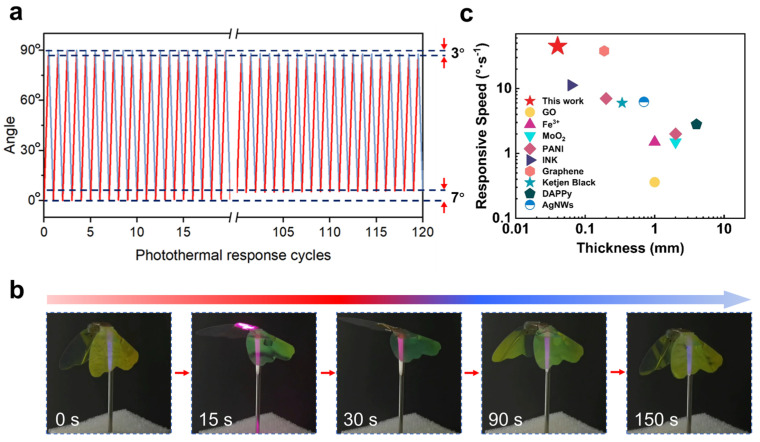
The application of NA/PET/NI-3 photothermal actuator. (**a**) Repeated testing cycles of the photothermal response driving for the NA/PET/NI-3 soft actuator film. The red lines represent conditions under near-infrared light irradiation, while the blue lines represent conditions with near-infrared light turned off. (**b**) The NA/PET/NI-3 soft actuator mimics the flapping motion of butterfly wings. (**c**) Comparison of this work with other photothermal-responsive soft actuators.

The repeatability of the NA/PET/NI-3 soft actuator was also observed as shown in Figure 4a. The NA/PET/NI-3 soft actuator was intermittently irradiated with NIR light, where we first turned on the light for 2 s to induce a bending response, followed by turning off the light to allow an eight-second recovery, and then the cycle was repeated. The results demonstrated that NA/PET/NI-3 could undergo photothermal response cycles for more than 120 times. The actuator bent quickly to 90° within 2 s, which was indicated by the red lines in the figure, and rapidly returned to its original shape within 8 s after the light was turned off, which was indicated by the blue lines in the figure. Nevertheless, after 120 cycles, the response angle decreased from the initial 90° to 83°, representing a reduction of approximately 7.8% in the response.

To demonstrate the photothermal properties of NA/PET/NI-3, a butterfly model was also designed by cutting the soft actuator into a rectangular shape of 10 × 20 mm^2^ with butterfly wing models attached to either side, as shown in Figure 4b. Under stimulation with near-infrared (NIR) light, the model flapped its wings in a manner similar to a butterfly. For the PNIPAM layer hydrogel, exposure to near-infrared (NIR) light induced a shrinking of the polymer chains on the side near the PET substrate, leading to a macroscopic bending of the PNIPAM hydrogel away from the light source. Similarly, the PNAGA layer hydrogel experienced chain swelling on the side adjacent to PET, causing the PNAGA hydrogel to bend macroscopically away from the light source. Combining the effects of bilateral polymers, the butterfly wings can be raised from a position perpendicular to the ground to a horizontal position within 30 s, which could be also observed on Video S1 in Supplementary Material in detail. The NA/PET/NI-3 could also be used in a grab application with small silicone balls, which could be wrapped and held when near-infrared light irradiated both ends of the actuator (Figure S5).

Furthermore, Figure 4c shows a comparison of the driving effects of soft polymer actuators containing different photothermal conversion materials, such as nanocarbon materials [33,34,35], metal particle [36,37,38], polyaniline (PANI) [39,40], ink [41], and dopamine decorated polypyrrole (DAPPy) [42]. The thicknesses of most soft actuators were in the millimeter range, whereas the soft actuator developed in this work was much thinner, with a thickness of 38.5 μm. To evaluate the photothermal response effect of the prepared NA/PET/NI-3, a response rate *V* was introduced as *V* = 90°/*t*. By measuring the time *t* it took for these actuators to bend to 90° under NIR light irradiation, the response rate was calculated for NA/PET/NI-3 and other photothermal-responsive soft actuators. The response rate of the soft actuator developed in this study reached 45°·s^−1^, which is faster than that of most other actuators, ranging from 0.36 to 38°·s^−1^. The results demonstrated that the three-layered structured soft film actuator (NA/PET/NI-3) utilized the synergistic effects of bilateral polymers, PNIPAM and PNAGA, on either side of PET. The micrometer thickness and bilateral polymer synergistic effect resulted in increasing the speed of the response.

## 4. Conclusions

In summary, this paper proposed a novel preparation strategy for a photothermal-responsive soft actuator based on carbon nanosheets of synergistic bilateral polymers. On the one hand, natural coconut water and PEI were used as the carbon sources to prepare carbon nanosheets with improved photothermal conversion capabilities through a simple method. On the other hand, by using the spin-coating method, the ultrathin film structure was constructed by NA/PET/NI-3 with a thickness of 38.5 μm, and the synergistic effect of LCST and UCST bilateral polymer properties significantly enhanced the response behavior of the soft actuator, bending to 90° within 2 s and recovering to its original shape within 8 s. This design provides new insights for the development of smart soft actuators.

## Data Availability

The original contributions presented in this study are included in the article/Supplementary Material. Further inquiries can be directed to the corresponding authors.

## Supplementary Materials

The following supporting information can be downloaded at: https://www.mdpi.com/article/10.3390/polym16243476/s1.
