# Supplementary material for: A Photothermal-Responsive Soft Actuator Based on Biomass Carbon Nanosheets of Synergistic Bilateral Polymers
Source: Polymers (Basel). 2024 Dec 13;16(24):3476. doi: 10.3390/polym16243476 (PMC11728592; doi:10.3390/polym16243476)
Supplement: Supplementary file 1 [file polymers-16-03476-s001.zip › polymers-3323520-supplementary.pdf]

polymers

MDPI

Article

A Photothermal-Responsive Soft Actuator Based on Biomass Carbon Nanosheets of Synergistic Bilateral Polymers

Jianze Chen [1,†], Quanzhong Wei [1,†], Honglin Wang [2], Wenjia Cui [1], Xuewei Zhang [1,*] and Yuanyuan Wang [3,*]

[1] Key Laboratory of Advanced Materials of Tropical Island Resources of Ministry of Education, School of Materials Science and Engineering, Hainan University, Haikou 570228, China; chjzemail@163.com (J.C.); 23220856010011@hainanu.edu.cn (Q.W.); 19935342032@163.com (W.C.)
[2] School of Tropical Agriculture and Forestry, Hainan University, Haikou 570228, China; wanghonglin0708@163.com
[3] NHC Key Laboratory of Tropical Disease Control, School of Tropical Medicine, Hainan Medical University, Haikou 571199, China
* Correspondence: zhangxuewei@hainanu.edu.cn (X.Z.); hy0105003@muhn.edu.cn (Y.W.)
† These authors contributed equally to this work.

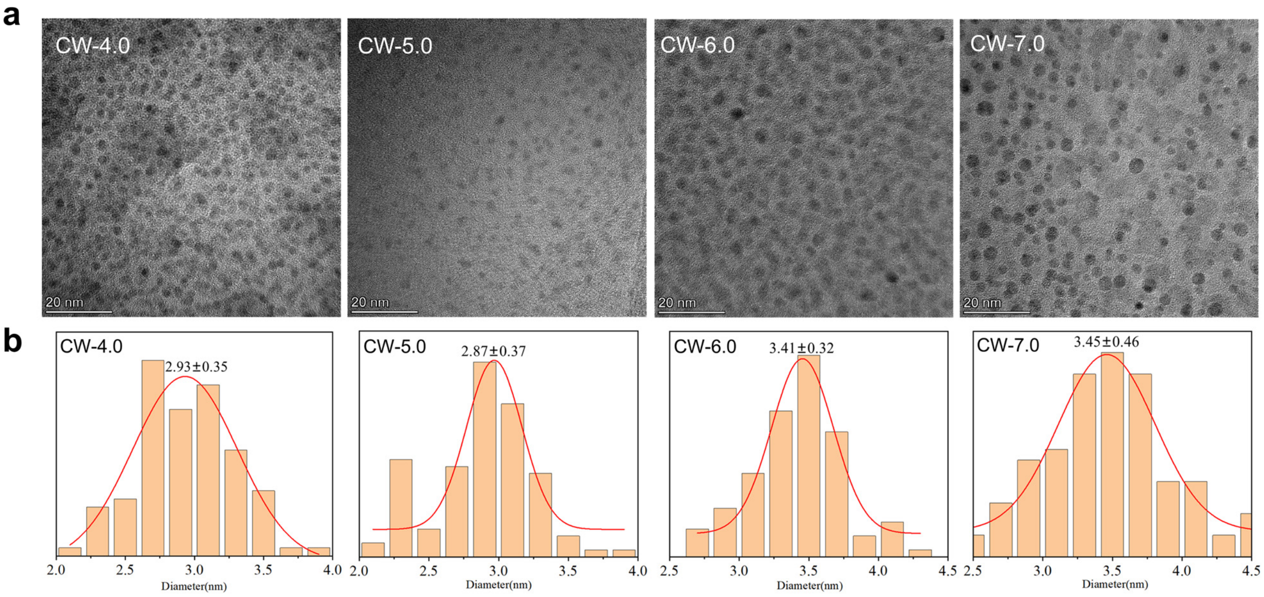

Figure S1. TEM of nanomaterials fabricated by coconut water though microwave carbonization method. (**a**) TEM with a microwave carbonization time of 4.0-7.0 minutes. The scale in the figure is 20 nm. (**b**) The particle size distribution of carbon dots prepared at different carbonization time.

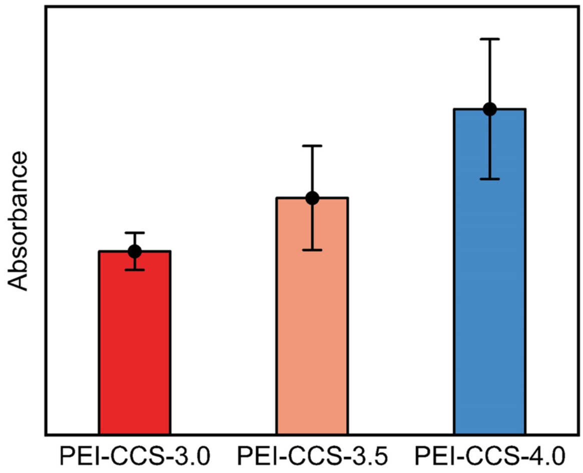

Figure S2. Absorbance of PEI-CCS solution with microwave carbonization time of 3.0, 3.5 and 4.0 min at wavelength of 808 nm.

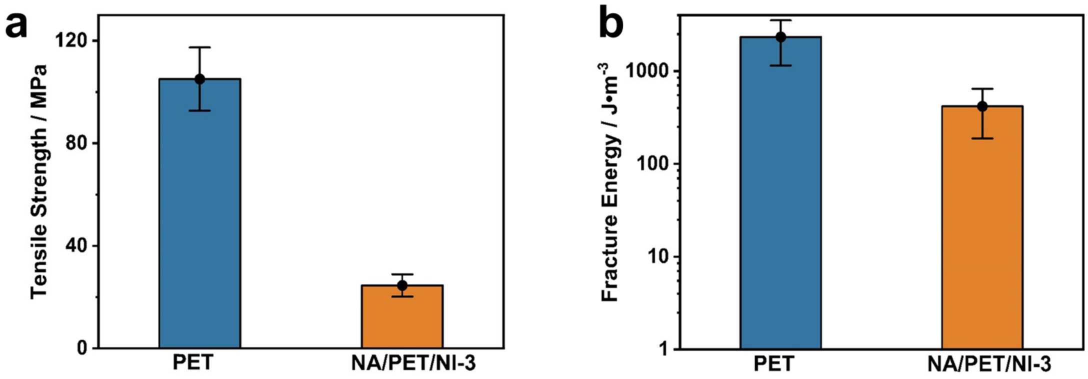

Figure S3. (**a**) Tensile fracture strength of PET film and NA/PET/NI-3 actuator. (**b**) Calculation results of fracture energy in the stress-strain curves of PET film and NA/PET/NI-3 film.

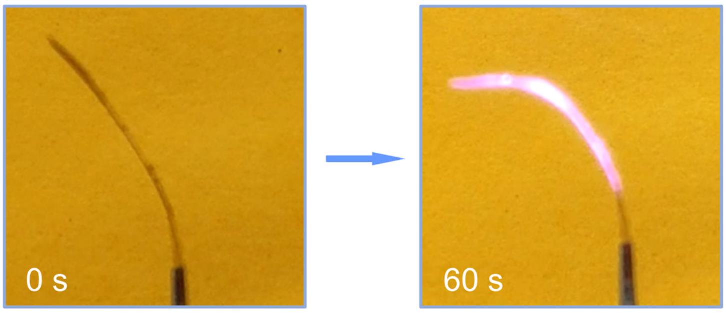

Figure S4. The photothermal response behavior of NI/PET soft actuator, in which the actuator only consisted of ten layers of PNIPAM, bending to 67° exposed to near-infrared light for 60 s.

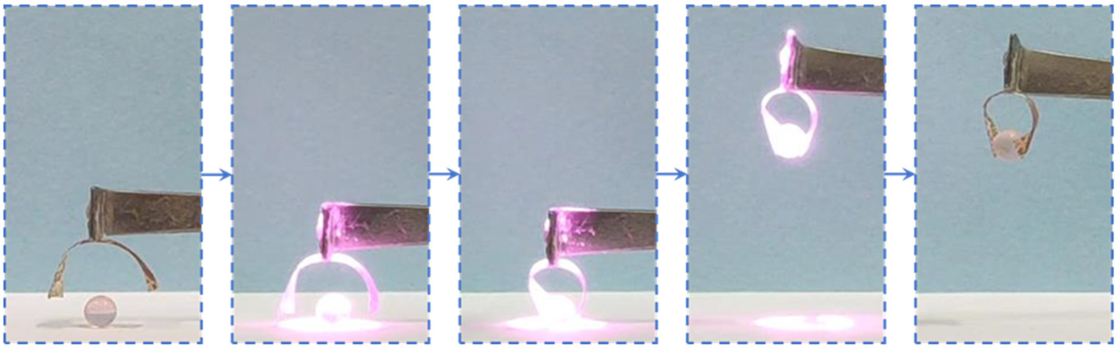

Figure S5. Grab-and-release application with small balls of the NA/PET/NI-3. A 50 mg silicone ball could be wrapped and held when near-infrared light irradiates both ends of the actuator.
